# Evaluating cystatin-C and monocyte-to-high-density lipoprotein cholesterol ratio as indicators of obstructive sleep apnea severity in male patients

**DOI:** 10.3389/fcvm.2025.1545100

**Published:** 2025-03-17

**Authors:** Run-Tian Meng, Qiao-Wen Chen, Chih-Yuan Ko

**Affiliations:** ^1^ Department of Clinical Nutrition, The Second Affiliated Hospital of Fujian Medical University, Quanzhou, Fujian, China; ^2^ The School of Public Health, Fujian Medical University, Fuzhou, Fujian, China

**Keywords:** hypertension, obstructive sleep apnea, cystatin-C, monocyte to high-density lipoprotein cholesterol ratio, obesity, inflammation

## Abstract

**Objectives:**

This study investigates the association between blood cystatin-C (Cys-C) and monocyte-to-high-density lipoprotein cholesterol ratio (MHR), both established inflammatory markers, with the severity of obstructive sleep apnea (OSA) in male patients.

**Methods:**

A total of 117 male participants who underwent overnight polysomnography (PSG) between February 2019 and December 2022 were included. Based on the apnea-hypopnea index (AHI), participants were categorized into three groups: G1 (AHI < 5 events/hour, *n* = 9; control group), G2 (5 ≤ AHI < 30 events/hour, *n* = 32), and G3 (AHI ≥ 30 events/hour, *n* = 76). Serum Cys-C and MHR levels were measured and analyzed for their correlation with OSA severity. Multivariate logistic regression and receiver operating characteristic (ROC) analyses assessed their diagnostic value, while restricted cubic spline (RCS) analysis examined potential nonlinear relationships.

**Results:**

Cys-C and MHR levels increased with OSA severity and showed significant positive correlations with AHI (Cys-C: *r* = 0.084, *P* < 0.05; MHR: *r* = 0.1286, *P* < 0.05). In multivariate regression, MHR remained an independent correlate of OSA severity (adjusted OR = 47.130, 95% CI: 1.014–6.692, *P* = 0.008), whereas Cys-C lost statistical significance after adjusting for confounders. RCS analysis found no significant nonlinear relationship (*P* > 0.05). ROC analysis showed that combining Cys-C and MHR modestly improved diagnostic accuracy (AUC = 0.6622, 95% CI: 0.554–0.77). Subgroup analysis indicated that severe OSA patients with hypertension had higher Cys-C and MHR levels compared to those without hypertension, though the differences were not statistically significant (*P* > 0.05).

**Conclusions:**

Cys-C and MHR are positively associated with OSA severity, with MHR emerging as a stronger independent biomarker. Incorporating these markers into OSA risk stratification may enhance clinical assessment and targeted interventions. Future large-scale prospective studies are needed to validate their prognostic value and clinical utility.

## 1 Introduction

Obstructive sleep apnea syndrome (OSA) is a common sleep-related breathing disorder characterized by recurrent upper airway partial or complete obstruction during sleep, leading to intermittent hypoxia and disrupted sleep architecture (1). With an estimated prevalence of 4% in China, OSA significantly impacts public health and socio-economic systems (2). The apnea-hypopnea index (AHI), derived from overnight polysomnography (PSG), is the gold standard for assessing OSA severity (3). However, PSG is resource-intensive and fails to capture the inflammatory and metabolic disturbances underlying OSA pathophysiology. The identification of accessible blood-based biomarkers may complement PSG, providing a more practical and efficient diagnostic approach.

The hallmark symptoms of OSA include snoring and apnea episodes, which, in severe cases, may lead to hypoxemia, carbon dioxide retention, respiratory failure, and pulmonary encephalopathy. In extreme scenarios, nocturnal hypoxia can result in sudden death (4). Chronic inflammation plays a central role in OSA pathophysiology, with intermittent hypoxia triggering systemic inflammation, oxidative stress, and endothelial dysfunction, thereby increasing cardiovascular risks (4, 5). Traditional inflammatory markers, such as C-reactive protein (CRP) and erythrocyte sedimentation rate, are well-established indicators but lack specificity in reflecting the complex inflammatory mechanisms of OSA. In contrast, cystatin-C (Cys-C) and the monocyte-to-high-density lipoprotein cholesterol ratio (MHR) may provide more targeted insights into OSA-associated inflammation (6, 7).

Cys-C, a cysteine protease inhibitor, is widely used as a sensitive biomarker of glomerular filtration rate and early renal impairment (8–10). Recent studies have highlighted its role in systemic inflammation and cardiovascular disease (11). Elevated serum Cys-C levels have been correlated with OSA severity and are independently associated with an increased risk of cardiovascular events and mortality (12). Moreover, Cys-C modulates protease hydrolysis, elevates blood homocysteine levels, and influences neutrophil migration and endothelial stability, thereby contributing to vascular inflammation and dysfunction in OSA (13).

MHR serves as an indicator of chronic vascular inflammation and is extensively studied in cardiovascular diseases (7). Monocytes and macrophages regulate inflammatory cytokine production and vascular remodeling, whereas high-density lipoprotein cholesterol exerts anti-inflammatory effects by suppressing monocyte activation and promoting cholesterol efflux (14). Elevated MHR levels have been associated with OSA severity, particularly in patients with hypertension (15), reflecting heightened systemic inflammation and oxidative stress (16). Both Cys-C and MHR are inflammatory biomarkers that contribute to cardiovascular disease and hypertension, conditions frequently exacerbated by OSA-related intermittent hypoxia and reoxygenation cycles (17, 18). These processes induce oxidative stress, endothelial dysfunction, and inflammatory responses, further worsening cardiovascular outcomes (19).

Although Cys-C and MHR have been investigated in relation to cardiovascular disease, their roles in OSA patients, particularly those with comorbid hypertension, remain incompletely understood. Given the cross-sectional design of this study, we emphasize that Cys-C and MHR serve as diagnostic markers of OSA severity rather than predictors of disease progression. Inflammatory biomarkers are crucial in OSA assessment, primarily for evaluating disease severity rather than establishing causal relationships.

The primary objective of this study is to examine the association between Cys-C, MHR, and OSA severity and to determine their potential as diagnostic biomarkers. This study seeks to establish whether these markers reflect the degree of systemic inflammation in OSA patients and their utility in identifying individuals at higher risk for comorbid conditions. By investigating the combined role of Cys-C and MHR in OSA, this study contributes to the understanding of inflammation-driven pathophysiological mechanisms and their clinical relevance. The findings may provide valuable insights into the potential applications of these biomarkers in risk stratification and disease management, thereby improving diagnostic approaches for OSA.

## 2 Material and methods

### 2.1 Participants

This study adheres to the principles of the Helsinki Declaration and has received approval from the Ethics Committee of the Second Affiliated Hospital of Fujian Medical University (IRB No. 2017-78 and 2020-160). A total of 117 male participants were enrolled in the study between February 2019 and December 2022. Written informed consent was obtained from all participants.

All participants underwent overnight PSG (SOMNOscreen™ plus PSG+, SOMNOmedics GmbH, Randersacker, Germany), which was conducted by trained technologists at the Sleep Medicine Center from 10 p.m.–8 a.m. The participants were newly diagnosed with OSA and had no prior history of medication treatment.

Exclusion criteria included participants with a history of OSA treatment, prior uvulopalatopharyngoplasty or continuous positive airway pressure therapy, antihypertensive medication use, active smoking, alcohol dependence, recent caffeine consumption or vigorous exercise, and those diagnosed with periodic limb movement disorder or severe insomnia. All participants underwent a comprehensive clinical evaluation to ensure eligibility.

Hypertension was defined as clinic blood pressure (BP) ≥140/90 mmHg, home BP ≥135/85 mmHg, or a 24 h ambulatory BP average of ≥130/80 mmHg. On the day of PSG monitoring, baseline characteristics were recorded, including age, average heart rate, blood pressure elevation index, average systolic blood pressure, and average diastolic blood pressure. Height and weight were measured to calculate body mass index (BMI). To ensure accuracy, all heart rate and blood pressure measurements were standardized and conducted in a quiet environment under controlled conditions.

### 2.2 PSG

All participants followed standardized preprocessing procedures before undergoing polysomnography (PSG) performed with a computerized polysomnographic system, to ensure the accuracy and consistency of the data. This included instructions for participants to avoid alcohol, caffeine intake, and strenuous exercise on the night before the monitoring, as these factors could affect sleep. All connections strictly followed the international 10–20 system, and scoring and analysis were conducted according to the AASM 2.6 version standards. PSG scoring was performed by professionally trained technicians to ensure data consistency and accuracy. On the evening of the examination, participants reported to the hospital and were fitted with various monitoring devices, including those for electroencephalography, electrocardiography, monitoring of eye movements and jaw muscles, thoracoabdominal respiratory movements, airflow through the nose and mouth, pulse oximetry, and snoring. A full-night PSG examination was conducted. The diagnostic results were analyzed and interpreted by the hospital's sleep technicians to calculate the apnea-hypopnea index. According to the diagnostic criteria established by the American Academy of Sleep Medicine in 2007, 3 all enrolled participants were categorized based on their AHI as follows: the G1 group (AHI < 5 events/hour, *n* = 9), as a control group; the G2 group (5 ≤ AHI < 30 events/hour, *n* = 32); and the G3 group (AHI ≥ 30 events/hour, *n* = 76). Considering that hypertension is a common complication of OSA and that OSA is an independent risk factor for hypertension, 15 the severe OSA group was further divided into two subgroups based on the presence or absence of a history of hypertension. These subgroups include severe OSA with hypertension (*n* = 44) and without hypertension (*n* = 32).

### 2.3 Blood biochemistry analysis

The morning following PSG monitoring, fasting venous blood samples were collected from all participants to conduct a comprehensive biochemical analysis. This analysis included measurements of cystatin-C (Cys-C) and additional inflammatory and metabolic biomarkers relevant to the study. Biochemical assessments were performed using the Mindray BS-280 fully automated biochemical analyzer (Mindray, Shenzhen, China), which ensured precision in measuring various parameters. The analyzer was calibrated prior to each testing session to maintain data accuracy and consistency. Specific assays were conducted for each biomarker, including enzymatic methods for lipid profiles and immunoturbidimetric methods for Cys-C, following manufacturer guidelines. Quality control samples were run alongside each batch to validate the reliability of results.

### 2.4 Blood cells analysis

Fasting venous blood samples were also collected to measure blood cell indices, including monocytes (MO) and high-density lipoprotein cholesterol (HDL-c). Blood cell evaluations were performed using the Mindray BC-7500 fully automated hematology analyzer (*N*MT, Shenzhen, China). The monocyte to high-density lipoprotein cholesterol ratio (MHR) was calculated based on the ratio of MO to HDL-c.

### 2.5 Statistical analysis

Statistical analyses were conducted using IBM SPSS Statistics software (version 29.0.1.0) and R software (version 4.1.1). Normally distributed data are presented as mean ± standard deviation (*X* ± SD), with intergroup comparisons performed using one-way analysis of variance (ANOVA) and independent samples *t*-test. Non-normally distributed data are reported as median and interquartile range P50, (P25, P75), analyzed using the Kruskal–Wallis (KW) test and Mann–Whitney *U* test. The analyses also included Spearman correlation analysis, simple linear regression and ordinal logistic regression. *P* values less than 0.05 were considered statistically significant. To robustly assess the associations between Cys-C, MHR, and severity OSA, the analyses adjusted for potential confounders such as age and BMI using a multivariate ordinal logistic regression framework. Considering the impact of renal function on Cys-C levels and the significant correlation of glomerular filtration rate with age, age was included as a critical covariate to control for its potential influence on the outcomes. The receiver operating characteristic (ROC) curve was utilized to calculate and compare the area under the curve (AUC), evaluating the diagnostic performance of Cys-C and MHR in assessing OSA severity. Additionally, restricted cubic spline (RCS) analysis was conducted to explore potential nonlinear relationships between Cys-C, MHR, and OSA severity.

## 3 Results

### 3.1 Characteristics of patients

As presented in Table 1, significant differences were observed among the groups in age, BMI, average heart rate, mean systolic blood pressure, and mean diastolic blood pressure (*P* < 0.05). The G1 group had a lower age compared to the G2 and G3 groups, while the G3 group exhibited a higher BMI and average heart rate than the G2 group. Additionally, pairwise comparisons of mean systolic and diastolic blood pressure among the three groups showed significant differences (*P* < 0.05), with both parameters increasing progressively with OSA severity.

**Table 1:** Characteristics, polysomnography (PSG), blood biochemistry, and blood cell of all subjects.

Index	G1(*n* = 9)	G2(*n* = 32)	G3(*n* = 76)	*P* value	G1 vs. G2	G1 vs. G3	G2 vs. G3
Characteristics							
Age (year)	23.00 ± 12.18	40.63 ± 12.87	41.13 ± 13.10	<0.001	**	**	ns
BMI (kg/m^2^)	18.93 (17.08, 25.44)	25.88 (23.62, 28.47)	30.07 (27.04, 32.2)	<0.001	ns	***	***
Average heart rate (times/min)	71.00 (61.50,88.00)	62.50 (58.25,74.25)	73.00 (65.00, 92.00)	0.012	ns	ns	*
Mean systolic blood pressure (mmHg)	103.67 ± 9.937	123.5 ± 13.877	131.08 ± 17.657	<0.001	***	***	*
Mean diastolic blood pressure (mmHg)	68.67 ± 4.848	81.75 ± 10.571	87.66 ± 12.624	<0.001	**	***	*
PSG							
Sleep efficiency (%)	77.10 (51.90, 81.20)	75.60 (60.68, 85.10)	82.60 (69.10, 88.60)	ns	ns	ns	ns
WASO (min)	102.90 (69.20, 164.80)	107.20 (62.13, 194.83)	75.80 (48.40, 147.50)	ns	ns	ns	ns
Arousal index (times/hour)	2.40 (1.40, 4.90)	2.95 (2.13, 4.08)	2.00 (1.20, 3.10)	0.029	ns	ns	*
N1 stage percentage (%)	20.90 (10.60, 29.05)	24.05 (19.13, 32.38)	34.30 (23.00, 45.60)	<0.001	ns	*	**
N2 stage percentage (%)	47.40 (36.30, 52.30)	47.55 (42.95, 53.68)	45.20 (33.70, 53.70)	ns	ns	ns	ns
N3 stage percentage (%)	17.10 (8.30, 24.30)	10.65 (7.93, 14.83)	3.80 (0, 9.70)	<0.001	ns	**	***
AHI (times/hour)	1.70 (0.60, 3.50)	16.75 (9.58, 23.30)	66.40 (52.40, 81.20)	<0.001	ns	***	***
Minimum SpO_2_ (%)	93.00 (85.50, 94.50)	85.00 (81.00, 88.75)	68.00 (64.00, 74.00)	<0.001	ns	***	***
Oxygen desaturation index (times/hour)	2.60 (0.85, 4.40)	17.30 (11.18, 24.10)	68.00 (54.80, 79.60)	<0.001	ns	***	***
SIT90 (%)	0 (0, 0.01)	0.01 (0.01, 0.05)	0.43 (0.22, 0.53)	<0.001	ns	***	***
SIT80 (%)	0 (0,0)	0 (0, 0)	0.07 (0.02, 0.16)	<0.001	ns	***	***
Blood biochemistry							
Cys-C (mg/L)	0.80 (0.74, 0.96)	0.86 (0.75, 0.95)	0.94 (0.85, 1.06)	0.026	ns	***	***
AST/ALT	1.15 (0.82,1.38)	0.83 (0.68, 0.98)	0.71 (0.54, 0.89)	0.002	ns	**	ns
GGT(U/L)	14.00 (9.95, 17.65)	28.25 (16.00, 41.78)	47.30 (24.60, 79.00)	<0.001	ns	***	**
TG (mmol/L)	0.87 (0.67, 1.51)	1.49 (1.18, 2.27)	1.86 (1.36, 2.72)	0.001	*	***	ns
UA (mmol/L)	343.11 ± 50.07	415.94 ± 74.77	445.49 ± 93.97	0.003	*	***	ns
Apo-B/Apo-A	0.58 ± 0.10	0.77 ± 0.19	0.96 ± 0.27	<0.001	*	***	***
BUN/CREA	0.08 (0.07, 0.09)	0.06 (0.05, 0.08)	0.06 (0.05, 0.07)	ns	ns	ns	ns
Blood cells							
MHR	0.25 (0.18, 0.35)	0.31 (0.20, 0.42)	0.36 (0.25, 0.52)	0.018	ns	*	*
RBC (×10^12^/L)	4.88 ± 0.24	5.02 ± 0.45	5.23 ± 0.39	0.007	ns	*	*
HGB (g/L)	144.0 (138.00, 148.00)	154.0 (145.25, 162.00)	160.0 (152.00, 166.00)	<0.001	ns	**	ns
HCT (L/L)	0.43 (0.42, 0.44)	0.45(0.43, 0.48)	0.47(0.45, 0.49)	<0.001	ns	***	ns

G1, AHI < 5 events/hour; G2, 5 ≤ AHI < 30 events/hour; G3, AHI ≥ 30 events/hour.

BMI, body mass index; WASO, wake after sleep onset; AHI, apnea-hypopnea index; SIT, supine index time; Cys-C, Cystatin-C; AST/ALT, aspartate aminotransferase/alanine aminotransferase; GGT, gamma-glutamyl transferase; TG, triglycerides; UA, uric acid; Apo-B/Apo-A, apolipoprotein B/apolipoprotein A; BUN/CREA, blood urea nitrogen/creatinine; MHR, monocyte-to-HDL cholesterol ratio; RBC, red blood cell; HGB, hemoglobin; HCT, hematocrit; ns, no statistical difference.

* *P* < 0.05.

** *P* < 0.01.

*** *P* < 0.001.

### 3.2 PSG

Comparisons of PSG-related indicators among the three groups revealed that the N1 stage percentage, AHI, oxygen desaturation index, SIT90, and SIT80 were significantly higher in the G3 group than in the G1 and G2 groups (*P* < 0.05). In contrast, the N3 stage percentage and minimum SpO₂ were lower in the G3 group compared to the G1 and G2 groups, with these differences also reaching statistical significance (*P* < 0.05). However, no significant differences were observed in other PSG-related indicators among the three groups (*P* > 0.05; Table 1).

### 3.3 Blood biochemistry and blood cells

In the comparison of blood indicators among the G1, G2, and G3 groups, patients in the G3 group exhibited significantly higher levels of Cys-C and GGT compared to those in the G1 and G2 groups. Additionally, TG, UA, and Apo-B/Apo-A levels were elevated in both the G2 and G3 groups relative to the G1 group, with statistically significant differences (*P* < 0.05; Table 1).

Patients in the G3 group also showed significantly higher levels of MHR, RBC, HGB, HCT, and RET compared to those in the G1 group. Moreover, MHR and RBC levels were higher in the G3 group than in the G2 group, with these differences reaching statistical significance (*P* < 0.05; Table 1).

### 3.4 Correlation results

**Figure 1:**
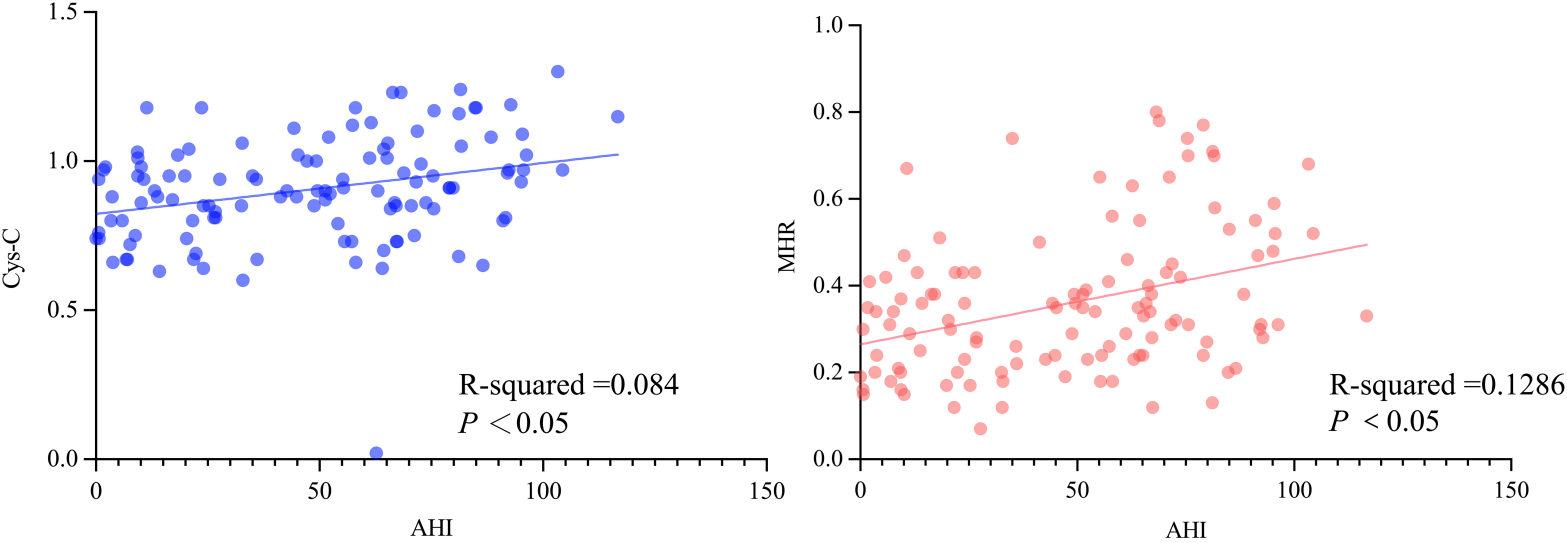
The correlation analysis between the apnea-hypopnea index (AHI) and cystatin-C (Cys-C) **(A)**, as well as the monocyte-to-high-density lipoprotein cholesterol ratio (MHR) **(B).**

Spearman correlation analysis and simple linear regression demonstrated a positive correlation between Cys-C and AHI (*r* = 0.316, *P* < 0.001), with the regression equation *Y* = 0.001708X + 0.8231 (*F* = 10.55, *R*² = 0.084, *P* < 0.05; Figure 1A). Similarly, MHR exhibited a positive correlation with AHI (*r* = 0.347, *P* < 0.001), with the regression equation *Y* = 0.001967X + 0.2651 (*F* = 16.97, *R*² = 0.1286, *P* < 0.05; Figure 1B). Through 1,000 bootstrap resampling iterations, the 95% confidence interval (CI) for the correlation coefficient between AHI and Cys-C was 0.137–0.475 (*P* < 0.001), confirming their association. Similarly, the 95% CI for the correlation coefficient between AHI and MHR was 0.171–0.501 (*P* < 0.001), further validating the correlation (Figure 1).

Spearman correlation analysis also assessed the relationship between Cys-C and multiple variables. Cys-C was positively correlated with AHI (*r* = 0.316, *P* < 0.001), N1 stage percentage (*r* = 0.202, *P* = 0.029), oxygen desaturation index (*r* = 0.291, *P* = 0.001), SIT90 (*r* = 0.286, *P* = 0.002), and SIT80 (*r* = 0.216, *P* = 0.019). A negative correlation was observed between Cys-C and minimum SpO₂ (*r* = −0.227, *P* = 0.014; Table 2). Similarly, MHR was positively correlated with AHI (*r* = 0.347, *P* < 0.001), oxygen desaturation index (*r* = 0.303, *P* < 0.001), SIT90 (*r* = 0.245, *P* = 0.008), and SIT80 (*r* = 0.262, *P* = 0.004). A negative correlation was observed between MHR and minimum SpO₂ (*r* = 0.285, *P* = 0.002; Table 2).

**Table 2:** The spearman correlation of cystatin-C (Cys-C) and monocyte-to-high-density lipoprotein cholesterol ratio (MHR) with polysomnography (PSG) parameters.

PSG-related parameters	Cys-C		MHR	
	R-squared	*P* value	R-squared	*P* value
Sleep efficiency (%)	0.034	ns	0.136	ns
WASO (min)	0.069	ns	−0.102	ns
Arousal index (times/hour)	0.069	ns	−0.161	ns
N1 stage percentage (%)	0.202	*	0.079	ns
N2 stage percentage (%)	−0.098	ns	0.107	ns
N3 stage percentage (%)	−0.0179	ns	−0.123	ns
AHI (times/hour)	0.316	***	0.347	***
minimum SpO_2_ (%)	−0.227	*	−0.285	**
oxygen desaturation index (times/hour)	0.291	***	0.303	***
SIT90 (%)	0.286	**	0.245	**
SIT80 (%)	0.216	*	0.262	**

WASO, wake after sleep onset; AHI, apnea-hypopnea index; SIT, supine index time; ns, no statistical difference.

* *P* < 0.05.

** *P* < 0.01.

*** *P* < 0.001.

### 3.5 Logistic regression results

The results of logistic regression analysis indicate that Cys-C is a risk factor for OSA severity. An increase in Cys-C levels is associated with a significant upward trend in OSA severity (*β* = 2.546, OR = 12.76, 95% CI: 0.391–4.701, *P* = 0.021). This suggests that for every 1-unit increase in Cys-C, the likelihood of OSA severity increasing by one grade is 12.76 times higher. After 1,000 bootstrap resampling verifications, the 95% CI for the regression coefficient of AHI and Cys-C was 0.479–6.006 (*P* < 0.001), further confirming their association.

Similarly, MHR was identified as an independent risk factor for OSA severity, with OSA severity progressively increasing with higher MHR levels (*β* = 4.226, OR = 68.44, 95% CI: 1.423–7.029, *P* = 0.003). Specifically, for every 1-unit increase in MHR, the likelihood of OSA severity increasing by one grade is 68.44 times higher. After 1,000 bootstrap resampling, the 95% CI for the regression coefficient of AHI and MHR was 2.006–7.000 (*P* < 0.001), reinforcing the association between AHI and MHR.

In multivariate ordinal logistic regression analysis, adjustments were made for potential confounders, including age and BMI. While the initial analysis demonstrated significant associations between Cys-C, MHR, and OSA severity, adjustments for age and BMI attenuated the effect of Cys-C, rendering it statistically non-significant (*β* = 1.944, OR = 6.99, 95% CI: −0.300–4.187, *P* = 0.089). Conversely, MHR remained a robust and statistically significant independent factor (*β* = 3.853, OR = 47.14, 95% CI: 1.014–6.692, *P* = 0.008). These findings suggest that after adjusting for age and BMI, MHR maintains a stronger and more independent association with OSA severity (Table 3).

**Table 3:** Logistic regression analysis of cystatin-C (Cys-C) and monocyte-to-high-density lipoprotein cholesterol ratio (MHR).

Logistic regression results	Index	*β*	OR	*P* value	CI 95%
Single factor logistic regression	Cys-C	2.546	12.760	0.021	0.391–4.701
	MHR	4.226	68.440	0.003	1.423–7.029
Multifactor logistic regression	Cys-C	1.944	6.990	0.089	−0.300–4.187
	MHR	3.853	47.130	0.008	1.014–6.692

### 3.6 Analysis of RCS

**Figure 2:**
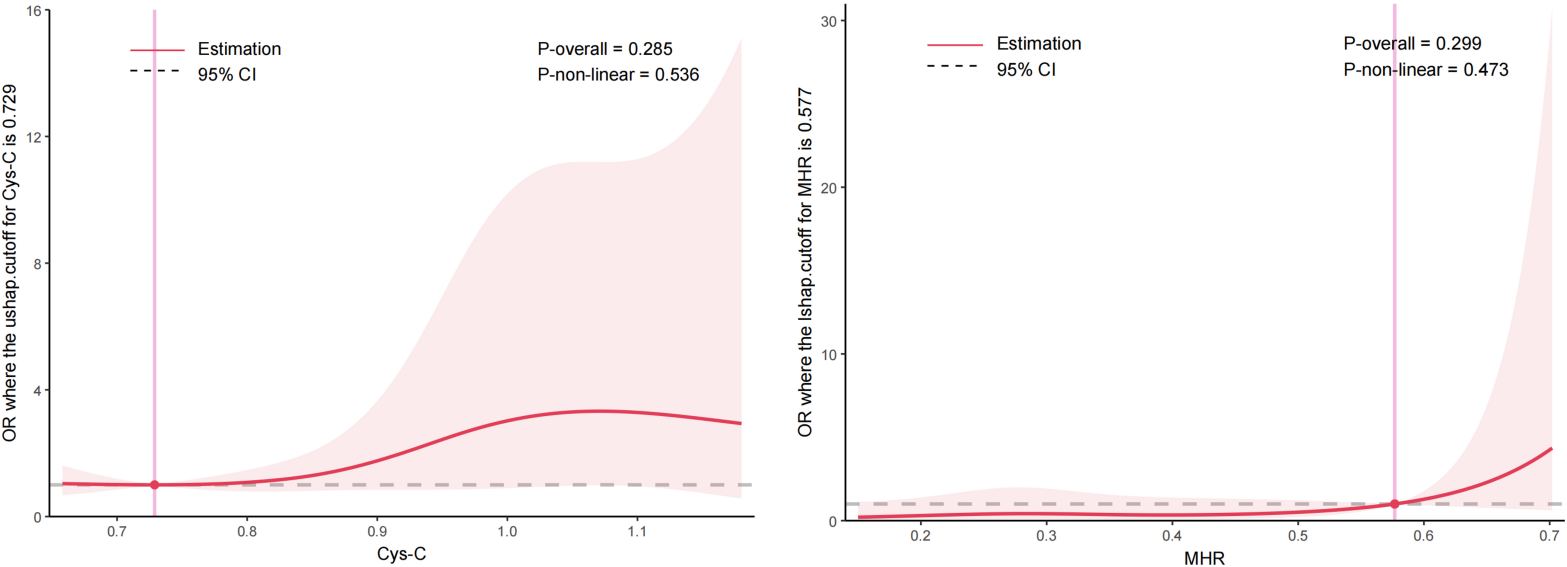
Non-linear relationships between cystatin-C (Cys-C) **(A)** and monocyte-to-high-density lipoprotein cholesterol ratio (MHR) **(B)** with AHI severity assessed by restricted cubic spline. The red lines represent the estimated effects, while the shaded areas denote the 95% confidence interval. Vertical lines indicate the threshold values for Cys-C (0.729) and MHR (0.577). The analysis revealed no significant non-linear relationships between these biomarkers and AHI severity.

RCS analysis indicated no significant nonlinear relationship between Cys-C, MHR, and AHI severity within the assessed ranges. Figure 2A shows the association between Cys-C and AHI severity, with no significant nonlinear effects (*P*-overall = 0.285, *P*-nonlinear = 0.536), suggesting a stable relationship across the observed range. Similarly, Figure 2B illustrates the association between MHR and AHI severity, where no significant nonlinear effects were observed (*P*-overall = 0.299, *P*-nonlinear = 0.473), indicating a consistent trend across the examined range.

### 3.7 Comparison of severe OSA patients with and without hypertension

**Figure 3:**
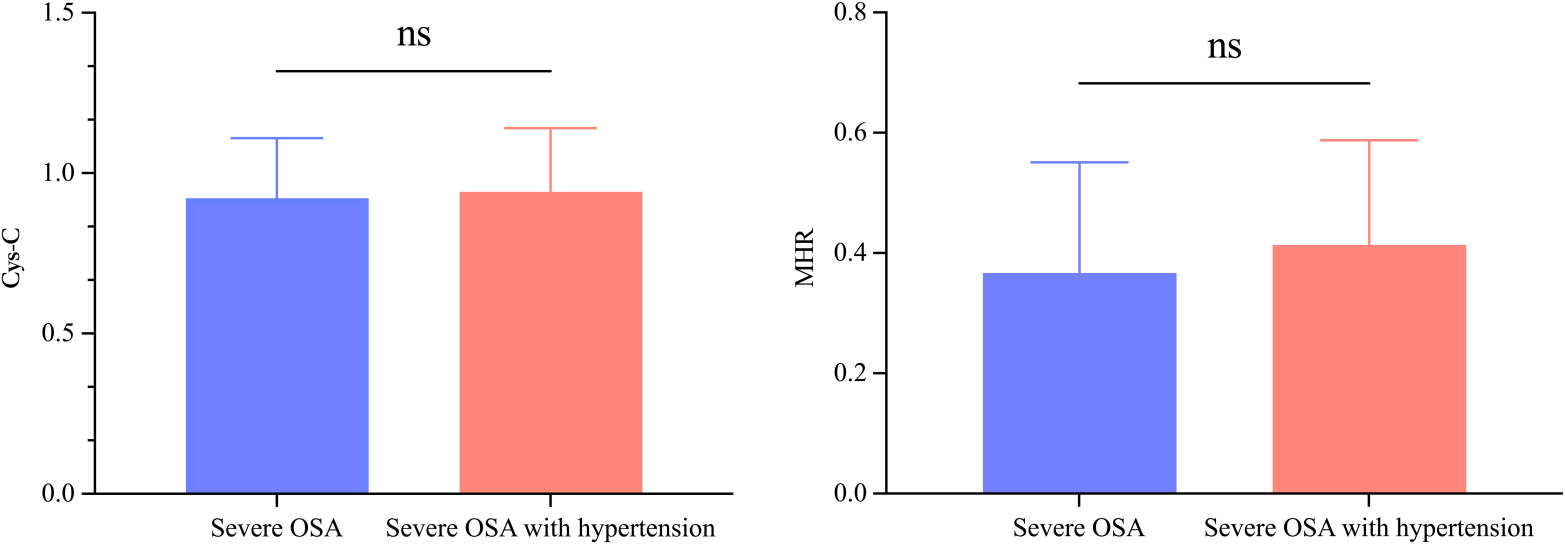
No significant differences were observed in cystatin-C (Cys-C) levels **(A)** or monocyte-to-high-density lipoprotein cholesterol ratio (MHR) levels **(B)** between severe obstructive sleep apnea (OSA) patients with and without hypertension. ns, No statistical difference.

In the comparison between severe OSA patients with and without hypertension, those with hypertension exhibited significantly higher mean systolic and diastolic blood pressures than those without hypertension (*P* < 0.05). However, no significant differences were observed in other clinical indicators between the two groups (Table 4). Regarding PSG parameters, patients with severe OSA and hypertension had higher sleep efficiency, oxygen desaturation index, and SIT80 compared to those without hypertension (*P* < 0.05). Conversely, arousal time after sleep onset and minimum oxygen saturation were lower in the severe OSA with hypertension group than in the non-hypertensive group (*P* < 0.05). No significant differences were found in blood biochemical and blood cell parameters between severe OSA patients with and without hypertension. However, Cys-C (0.86 ± 0.12 vs. 0.99 ± 0.15, *P* > 0.05) and MHR (0.25 ± 0.13 vs. 0.42 ± 0.19, *P* > 0.05) exhibited an increasing trend in the hypertensive group (Figure 3).

**Table 4:** Characteristics, polysomnography (PSG), blood biochemistry, and blood cell of the severe obstructive sleep apnea (OSA) patients.

Index	Severe OSA (*n* = 32)	Severe OSA with hypertension (*n* = 44)	*P* value
Characteristics			
Age (year)	40.94 ± 14.91	41.27 ± 11.80	0.700
BMI (kg/m^2^)	28.41 (25.95, 32.53)	30.58 (27.90, 31.62)	0.305
Average heart rate (times/min)	72.00 (65.00, 91.00)	73.00 (64.50, 93.75)	0.755
Mean systolic blood pressure (mmHg)	121.00 (111.00, 129.00)	134.00 (126.00, 149.50)	<0.001
Mean diastolic blood pressure (mmHg)	82.00 (74.00, 87.00)	92.00 (86.25, 99.75)	<0.001
PSG			
Sleep efficiency (%)	76.20 (65.20, 82.60)	87.00 (74.25, 88.90)	0.015
WASO (min)	107.00 (50.90, 171.40)	62.05 (45.93, 115.55)	0.034
Arousal index (times/hour)	2.20 (1.40, 3.70)	1.90 (1.03, 2.68)	0.077
N1 stage percentage (%)	40.76 ± 27.25	36.96 ± 17.89	0.466
N2 stage percentage (%)	46.88 ± 23.74	43.17 ± 14.72	0.405
N3 stage percentage (%)	4.10 (1.00,9.30)	3.55 (0,12.10)	0.903
AHI (times/hour)	65.10 (51.30, 73.80)	68.00 (57.55, 85.30)	0.176
Minimum SpO_2_ (%)	71.00 (66.00, 77.00)	67.00 (62.00, 74.00)	0.031
Oxygen desaturation index (times/hour)	61.59 ± 19.26	70.80 ± 17.96	0.036
SIT90 (%)	0.35 (0.22, 0.46)	0.44 (0.22, 0.58)	0.066
SIT80 (%)	0.05 (0.02, 0.09)	0.09 (0.03, 0.22)	0.027
Blood biochemistry			
Cys-C (mg/L)	0.89 (0.75, 1.08)	0.96 (0.89, 1.06)	0.312
AST/ALT	0.79 ± 0.30	0.69 ± 0.21	0.126
GGT(U/L)	42.30 (22.00, 74.00)	48.50 (25.50, 80.50)	0.310
TG (mmol/L)	1.71 (1.23, 2.52)	2.00 (1.51, 2.79)	0.312
UA (mmol/L)	435.00 ± 81.58	453.11 ± 102.29	0.410
Apo-B/Apo-A	0.95 ± 0.29	0.96 ± 0.26	0.884
BUN/CREA	0.06 (0.05, 0.08)	0.06 (0.05, 0.07)	0.651
Blood cells			
MHR	0.34 (0.23,0.43)	0.37 (0.27,0.55)	0.234
RBC (×10^12^/L)	5.14 ± 0.40	5.29 ± 0.38	0.097
HGB (g/L)	157.00 (148.00, 163.00)	161.50 (152.00, 166.75)	0.106
HCT (L/L)	0.46(0.44, 0.49)	0.47(0.45, 0.50)	0.145

BMI, body mass index; WASO, wake after sleep onset; AHI, apnea-hypopnea index; SIT, supine index time; Cys-C, Cystatin-C; AST/ALT, aspartate aminotransferase/alanine aminotransferase; GGT, gamma-glutamyl transferase; TG, triglycerides; UA, uric acid; Apo-B/Apo-A, apolipoprotein B/apolipoprotein A; BUN/CREA, blood urea nitrogen/creatinine; MHR, monocyte-to-HDL cholesterol ratio; RBC, red blood cell; HGB, hemoglobin; HCT, hematocrit.

### 3.8 Receiver operating characteristic (ROC) curve

**Figure 4:**
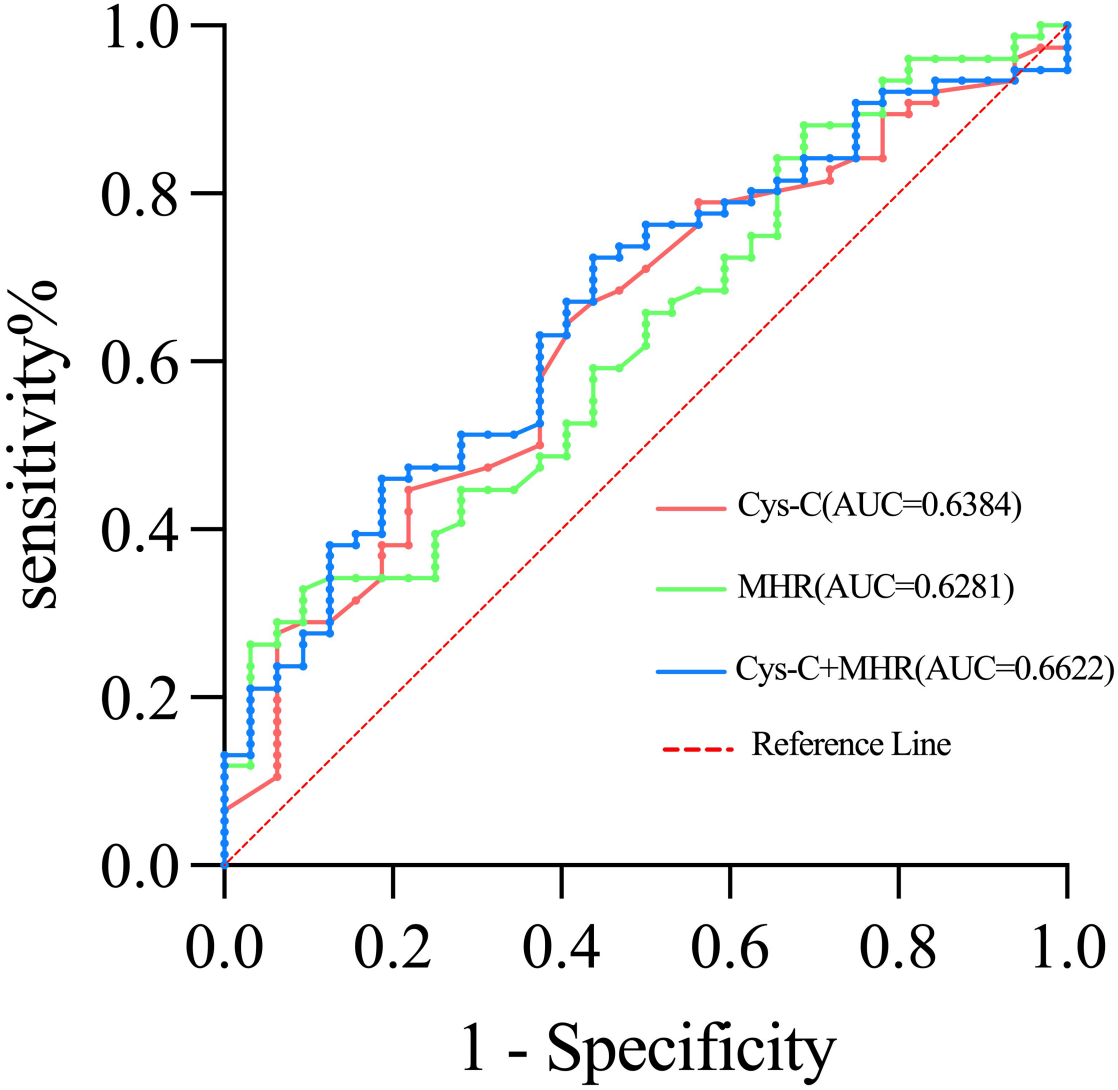
Receiver operating characteristic (ROC) curves for cystatin-C (Cys-C) and monocyte-to-high-density lipoprotein cholesterol ratio (MHR).

ROC curve analysis was performed to assess the diagnostic value of Cys-C and MHR in relation to OSA severity. The area under the curve (AUC) for Cys-C was 0.638, with a sensitivity of 64.5% and a specificity of 59.4%. For MHR, the AUC was 0.628, with a sensitivity of 62.8% and a specificity of 90.6%. When both biomarkers were combined, the AUC improved to 0.662, with an increased sensitivity of 72.4% but a lower specificity of 56.2% (Figure 4, Table 5).

**Table 5:** Predictive value of cystatin-C (Cys-C) and monocyte-to-high-density lipoprotein cholesterol ratio (MHR) in determining patient with OSA.

Index	AUC	Sensitivity	Specificity	Cutoff value	CI 95%	*P* value
Cys-C	0.6384	0.645	0.594	0.885	0.527–0.750	0.015
MHR	0.6281	0.329	0.906	0.442	0.516–0.740	0.025
Cys-C + MHR	0.6622	0.724	0.562	0.653	0.554–0.770	0.003

AUC, area under the ROC curve; Cl, confidence interval.

## 4 Discussion

This study demonstrated elevated levels of Cys-C and MHR in OSA patients, with the highest levels observed in severe cases. Both biomarkers showed a positive correlation with AHI, which was further validated through bootstrap resampling. While Cys-C and MHR exhibited potential as auxiliary diagnostic markers for OSA, only MHR remained a significant independent association after adjusting for age and BMI, reinforcing its reliability in assessing OSA severity. However, neither marker demonstrated sufficient sensitivity to severe OSA in hypertensive patients.

Inflammation plays a multifaceted role in OSA, with recurrent hypoxia during sleep triggering systemic immune activation, leading to increased inflammatory cytokines and immune cell activity (4, 7, 18). These inflammatory responses contribute to endothelial dysfunction and atherosclerosis, underlying the cardiovascular complications commonly observed in OSA patients. The biomarkers examined in this study, Cys-C and MHR, serve as indicators of this inflammatory burden, potentially reflecting disease severity.

Traditionally recognized as a sensitive kidney function biomarker, Cys-C is also involved in vascular function and inflammation (20, 21). Studies have reported that Cys-C levels correlate with OSA severity and oxygen desaturation, even in the absence of chronic kidney disease (22). Furthermore, Cys-C levels tend to decrease following CPAP treatment, suggesting its potential role in cardiovascular risk stratification (23). Additionally, Cys-C interacts with homocysteine, amplifying endothelial dysfunction and oxidative stress, particularly in hypertensive OSA patients (24–26), underscoring its relevance in OSA, hypertension, and cardiovascular health.

MHR reflects the balance between monocyte-driven inflammation and HDL-mediated anti-inflammatory effects, providing insights into systemic inflammation in OSA. Elevated MHR levels strongly correlate with OSA severity, positioning it as a promising marker for disease progression (22, 27). Beyond OSA, MHR is recognized as an inflammatory marker associated with major cardiovascular risks, cardiac events, and hypertensive end-organ damage (15, 28). These findings align with existing research, highlighting the central role of inflammation in OSA pathogenesis. As OSA severity progresses, Cys-C and MHR levels increase, suggesting their potential role in reflecting disease progression. Combining these markers may provide a more comprehensive diagnostic approach.

OSA is a well-established risk factor for hypertension (18). In this study, severe OSA patients with hypertension exhibited a non-significant increasing trend in Cys-C and MHR levels compared to those without hypertension. This discrepancy may be attributed to the small sample size, limiting statistical power, as well as unadjusted confounding factors such as individual inflammatory responses and vascular function variability. Intermittent hypoxia in OSA exacerbates oxidative stress and endothelial inflammation, particularly in hypertensive patients, potentially contributing to elevated Cys-C and MHR levels (15). Larger-scale studies are warranted to further establish their role as biomarkers for inflammation and oxidative stress in severe OSA with hypertension, thereby enhancing their clinical utility.

The role of Cys-C as an inflammatory and cardiovascular biomarker remains complex. Elevated Cys-C levels have been associated with inflammation in atherosclerosis and vulnerable plaque formation, while low levels have been linked to impaired infection resistance and chronic low-grade inflammation, potentially contributing to plaque progression (29, 30). Experimental studies suggest that Cys-C deficiency enhances cysteine protease activity, triggering vascular remodeling and inflammation (Ref. 31). Conversely, elevated Cys-C levels may represent a compensatory response to acute inflammation, which normalizes in chronic conditions (32, 33). The weaker correlation of Cys-C with OSA severity in this study, compared to MHR, may reflect these intricate regulatory dynamics. MHR, as a more robust inflammation marker, demonstrated stronger associations with OSA severity, particularly in patients with coexisting cardiovascular risks, reinforcing its clinical relevance (34).

Beyond its well-established impact on cardiovascular health, inflammation has also been increasingly recognized for its role in other physiological processes, including bone metabolism. Recent studies have identified the systemic inflammation response index as a composite inflammatory marker associated with both cardiovascular disease and bone health, suggesting a broader involvement of inflammation in disease pathogenesis (35). In OSA, chronic low-grade inflammation, driven by recurrent hypoxia and oxidative stress, may not only exacerbate hypertension, atherosclerosis, and heart failure but also impair bone metabolism, potentially increasing the risk of osteoporosis and fractures.

While, obesity is a major contributor to OSA severity, as it promotes airway narrowing, increases upper airway collapse risk, and exacerbates intermittent hypoxia, worsening disease progression (36). Recent studies have identified alternative obesity indices, such as waist-to-weight index (37) and body roundness index (38), which have also been associated with adverse OSA outcomes. Beyond mechanical airway obstruction, obesity is linked to chronic low-grade inflammation, potentially contributing to elevated Cys-C and MHR levels and increasing cardiovascular risk (39). These findings underscore the importance of considering obesity-related factors when assessing OSA severity and associated complications. To minimize BMI-related confounding effects, we adjusted for BMI in multivariate analyses, and MHR remained an independent marker of OSA severity, whereas Cys-C lost statistical significance, reinforcing MHR serves as a more robust biomarker of OSA-related inflammation, independent of obesity-related influences, reinforcing its potential clinical utility in assessing disease severity.

Other inflammatory markers, including neutrophil-to-lymphocyte ratio, platelet-to-lymphocyte ratio, eosinophil-to-lymphocyte ratio, monocyte-to-lymphocyte ratio, and systemic immune-inflammation index, were also analyzed in this study. However, due to a lack of statistical significance, these results were not presented. Previous research suggests that these ratios are commonly used as inflammatory biomarkers to assess OSA diagnostic value and severity indicators (40). Further research is needed to clarify their roles in pathophysiology of OSA.

This study has several limitations. The small sample size necessitates validation in larger cohorts to ensure greater statistical power and reproducibility. Additionally, the cross-sectional design precludes causal inference, making it unclear whether changes in Cys-C and MHR contribute to OSA progression or simply reflect disease characteristics. The underlying regulatory mechanisms of Cys-C and MHR in OSA and their potential effects on blood pressure regulation were not explored. Despite adjustments for known variables, unmeasured confounders may still influence the results.

Future longitudinal studies are needed to assess temporal changes in Cys-C and MHR, clarify their causal relationships with OSA severity, and evaluate their predictive value for OSA progression and cardiovascular outcomes. Moreover, incorporating a broader range of clinical and laboratory variables would provide a more comprehensive understanding of OSA pathophysiology. Given the well-established role of obesity in OSA, future research should stratify participants based on obesity levels to better delineate how Cys-C and MHR influence OSA severity across different obesity phenotypes. While this study demonstrates the associations between Cys-C, MHR, and OSA severity, these biomarkers should not be solely relied upon for clinical decision-making. Further research should explore their mechanistic roles in OSA-related inflammation and cardiovascular dysfunction, as well as investigate potential therapeutic interventions targeting these pathways. Expanding sample sizes and conducting multicenter studies will enhance the generalizability and clinical relevance of these findings.

These results highlight the potential of Cys-C and MHR as biomarkers for assessing OSA severity and cardiovascular risk. Integrating these markers into clinical practice may improve early identification of high-risk patients and facilitate targeted interventions to reduce cardiovascular complications and improve patient outcomes.

## 5 Conclusion

This single-center cross-sectional study highlights the significant association between Cys-C, MHR, and OSA severity, with both biomarkers increasing progressively with disease severity and exhibiting positive correlations with AHI. While Cys-C showed potential as an OSA severity correlate, MHR emerged as a more robust independent marker, maintaining significance after adjusting for confounders such as age and BMI. The combined assessment of Cys-C and MHR further improved diagnostic accuracy, suggesting their complementary roles in capturing the inflammatory and cardiovascular implications of OSA. However, their ability to distinguish between severe OSA patients with and without hypertension remains limited, warranting further investigation. Future large-scale, longitudinal, and multicenter studies are essential to validate these findings, refine their clinical applicability, and establish their prognostic value in OSA management and cardiovascular risk assessment, ultimately contributing to improved patient outcomes.

## Data Availability

The original contributions presented in the study are included in the article/Supplementary Material, further inquiries can be directed to the corresponding author.

## References

[1] Young T Peppard PE Gottlieb DJ. Epidemiology of obstructive sleep apnea: a population health perspective. Am J Respir Crit Care Med. (2002) 165(9):1217–39. 10.1164/rccm.2109080 11991871

[2] Senaratna CV Perret JL Lodge CJ Lowe AJ Campbell BE Matheson MC Prevalence of obstructive sleep apnea in the general population: a systematic review. J Sleep Med Rev. (2017) 34:70–81. 10.1016/j.smrv.2016.07.002 27568340

[3] Kapur VK Auckley DH Chowdhuri S Kuhlmann DC Mehra R Ramar K Clinical practice guideline for diagnostic testing for adult obstructive sleep apnea: an American academy of sleep medicine clinical practice guideline. JCSM Off Publ Am Acad Sleep Med. (2017) 13(3):479–504. 10.5664/jcsm.6506 PMC5337595 28162150

[4] Locke BW Lee JJ Sundar KM. OSA and chronic respiratory disease: mechanisms and epidemiology. Int J Environ Res Public Health. (2022) 19(9):5473. 10.3390/ijerph19095473 35564882 PMC9105014

[5] Baltzis D Bakker JP Patel SR Veves A. Obstructive sleep apnea and vascular diseases. Compr Physiol. (2016) 6(3):1519–28. 10.1002/cphy.c150029 27347900

[6] Ganjali S Gotto AM Jr Ruscica M Atkin SL Butler AE Banach M Monocyte-to-HDL-cholesterol ratio as a prognostic marker in cardiovascular diseases. J Cell Physiol. (2018) 233(12):9237–46. 10.1002/jcp.27028 30076716

[7] Evangelopoulos AA Vallianou NG Bountziouka V Katsagoni C Bathrellou E Vogiatzakis ED Association between serum cystatin C, monocytes and other inflammatory markers. Intern Med J. (2012) 42(5):517–22. 10.1111/j.1445-5994.2011.02500.x 21470355

[8] Clausen J. Proteins in normal cerebrospinal fluid not found in serum.. Proc Soc Exp Biol Med. (1961) 107:170–2. 10.3181/00379727-107-26569 13693957

[9] Simonsen O Grubb A Thysell H. The blood serum concentration of cystatin C (gamma-trace) as a measure of the glomerular filtration rate. Scand J Clin Lab Invest. (1985) 45(2):97–101. 10.3109/00365518509160980 3923607

[10] Wang RR He M Gui X Kang Y. A nomogram based on serum cystatin C for predicting acute kidney injury in patients with traumatic brain injury. Renal Fail. (2021) 43(1):206–15. 10.1080/0886022X.2021.1871919 PMC7833079 33478333

[11] Salgado JV Souza FL Salgado BJ. How to understand the association between cystatin C levels and cardiovascular disease: imbalance, counterb alance, or consequence? J Cardiol. (2013) 62(6):331–5. 10.1016/j.jjcc.2013.05.015 23849291

[12] Li JH Gao YH Xue X Su XF Wang HH Lin JL Association between Serum cystatin C levels and long-term cardiovascular outcomes and all-cause mortality in older patients with obstructive sleep apnea. Front Physiol. (2022) 13:934413. 10.3389/fphys.2022.934413 36117703 PMC9471320

[13] Ruiz SA Cortes RM Alegre BN Algarra-Garcia J de Teresa Galvan E Jimenez-Navarro MF. Relationship between cystatin C and coronary artery calcification in patients with intermediate cardiovascular risk. Med Clin. (2014) 143(8):535–8. 10.1016/j.medcli.2013.10.034 24725853

[14] Zhang Y Li S Guo YL Wu NQ Zhu CG Gao Y Is monocyte to HDL ratio superior to monocyte count in predicting the cardiovascular outcomes: evidence from a large cohort of Chinese patients undergoing coronary angiography. Ann Med. (2016) 48(5):305–12. 10.3109/07853890.2016.1168935 27087382

[15] Sun M Liang C Lin H Meng Y Tang Q Shi X Monocyte to HDL cholesterol ratio as a marker of the presence and severity of obstructive sleep apnea in hypertensive patients. Sci Rep. (2021) 11(1):15821. 10.1038/s41598-021-95095-3 34349139 PMC8338958

[16] Cetin MS Ozcan Cetin EH Kalender E Aydin S Topaloglu S Kisacik HL Monocyte to HDL cholesterol ratio predicts coronary artery disease severity and future major cardiovascular adverse events in acute coronary syndrome. Heart Lung Circ. (2016) 25(11):1077–86. 10.1016/j.hlc.2016.02.023 27118231

[17] Hou H Zhao Y Yu W Dong H Xue X Ding J Association of obstructive sleep apnea with hypertension: a systematic review and meta-analysis. J Glob Health. (2018) 8(1):010405. 10.7189/jogh.08.010405 29497502 PMC5825975

[18] Geovanini GR Wang R Weng J Jenny NS Shea S Allison M Association between obstructive sleep apnea and cardiovascular risk factors: variation by age, sex, and race. The multi-ethnic study of atherosclerosis. Ann Am Thorac Soc. (2018) 15(8):970–7. 10.1513/AnnalsATS.201802-121OC 29742365 PMC6322035

[19] Libby P. Inflammation in atherosclerosis-No longer a theory. J Clin Chem. (2021) 67(1):131–42. 10.1093/clinchem/hvaa275 33393629

[20] Newman DJ. Cystatin C. Ann Clin Biochem. (2002) 39(Pt 2):89–104. 10.1258/0004563021901847 11928770

[21] Chuang LP Lin SW Lee LA Chang CH Huang HY Hu HC Elevated serum markers of acute kidney injury in patients with obstructive sleep apnea. J Clin Sleep Med. (2019) 15(2):207–13. 10.5664/jcsm.7618 30736871 PMC6374082

[22] Kato K Takata Y Usui Y Shiina K Asano K Hashimura Y Severe obstructive sleep apnea increases cystatin C in clinically latent renal dysfunction. Respir Med. (2011) 105(4):643–9. 10.1016/j.rmed.2010.11.024 21183327

[23] Zhang XB Jiang XT Lin QC Chen X Zeng HQ. Effect of continuous positive airway pressure on serum cystatin C among obstructive sleep apnea syndrome patients. Int Urol Nephrol. (2014) 46(10):1997–2002. 10.1007/s11255-014-0779-x 25000895

[24] Gao Y Guo Y Hao W Meng J Miao Z Hou A Correlation analysis and diagnostic value of serum homocysteine, cystatin C and uric acid levels with the severity of coronary artery stenosis in patients with coronary heart disease. Int J Gen Med. (2023) 16:2719–31. 10.2147/IJGM.S411417 37405124 PMC10317548

[25] Chen ZH Zhu XT Hu ZP Ni JX Chen HL. Correction: correlation of serum homocysteine and cystatin C levels with prognosis in heart failure with preserved ejection fraction patients. BMC Cardiovasc Disord. (2024) 24(1):510. 10.1186/s12872-024-04058-9 39327565 PMC11428330

[26] Zhao PQ Li XY Jia W Dong BB Yang QT Wu C. Risk factors of serum cystatin-C in patients with hypertension combined with obstructive sleep apnea. Int J Cardiovasc Dis. (2016) 43(06):383–6. 10.3969/j.issn.1673-6583.2016.06.017

[27] Wu TT Zheng YY Chen Y Yu ZX Ma YT Xie X. Monocyte to high-density lipoprotein cholesterol ratio as long-term prognostic marker in patients with coronary artery disease undergoing percutaneous coronary intervention. Lipids Health Dis. (2019) 18(1):180. 10.1186/s12944-019-1116-2 31640740 PMC6805452

[28] Selcuk M Yildirim E Saylik F. Comparison of monocyte with high density lipoprotein cholesterol ratio in dipper and nondipper hypertensive patients. Biomark Med. (2019) 13(15):1289–96. 10.2217/bmm-2019-0062 31596122

[29] Li YY Gao XL. Serum predictors of obstructive sleep apnea syndrome combined with cardiovascular disease. Chin J Geriatric Multi-Organ Dis. (2002) 21(11):854–7. 10.11915/j.issn.1671-5403.2022.11.183

[30] Shi GP Sukhova GK Grubb A Ducharme A Rhode LH Lee RT Cystatin C deficiency in human atherosclerosis and aortic aneurysms. J Clin Invest. (1999) 104(9):1191–7. 10.1172/JCI7709 10545518 PMC409823

[31] Schulte S Sun J Libby P MacFarlane L Sun C Lopez-Ilasaca M Cystatin C deficiency promotes inflammation in angiotensin II-induced abdominal aortic aneurisms in atherosclerotic mice. Am J Pathol. (2010) 177(1):456–63. 10.2353/ajpath.2010.090381 20472891 PMC2893687

[32] Gu FF Lü SZ Chen YD Zhou YJ Song XT Jin ZN Relationship between plasma cathepsin S and cystatin C levels and coronary plaque morphology of mild to moderate lesions: an *in vivo* study using intravascular ultrasound. Chin Med J. (2009) 122(23):2820–6. 10.3760/cma.j.issn.0366-6999.2009.23.004 20092784

[33] Doganer YC Aydogan U Aydogdu A Aparci M Akbulut H Nerkiz P Relationship of cystatin C with coronary artery disease and its severity. Coron Artery Dis. (2013) 24(2):119–26. 10.1097/MCA.0b013e32835b6761 23211477

[34] Li N Ren L Wang JH Yan YR Lin YN Li QY. Relationship between monocyte to HDL cholesterol ratio and concomitant cardiovascular disease in Chinese Han patients with obstructive sleep apnea. Cardiovasc Diagn Ther. (2019) 9(4):362–70. 10.21037/cdt.2019.08.02 31555541 PMC6732074

[35] Ma H Cai X Hu J Song S Zhu Q Zhang Y Association of systemic inflammatory response index with bone mineral density, osteoporosis, and future fracture risk in elderly hypertensive patients. Postgrad Med. (2024) 136(4):406–16. 10.1080/00325481.2024.2354158 38753519

[36] Peppard PE Young T Palta M Dempsey J Skatrud J. Longitudinal study of moderate weight change and sleep-disordered breathing. JAMA. (2000) 284(23):3015–21. 10.1001/jama.284.23.3015 11122588

[37] Zhao J Cai X Hu J Song S Zhu Q Shen D J-shaped relationship between weight-adjusted-waist index and cardiovascular disease risk in hypertensive patients with obstructive sleep apnea: a cohort study. Diabetes Metab Syndr Obes. (2024) 17:2671–81. 10.2147/DMSO.S469376 38978818 PMC11228610

[38] Cai X Song S Hu J Zhu Q Yang W Hong J Body roundness index improves the predictive value of cardiovascular disease risk in hypertensive patients with obstructive sleep apnea: a cohort study. Clin Exp Hypertens. (2023) 45(1):2259132. 10.1080/10641963.2023.2259132 37805984

[39] Wei Z Chen Y Upender RP. Sleep disturbance and metabolic dysfunction: the roles of adipokines. Int J Mol Sci. (2022) 23(3):1706. 10.3390/ijms23031706 35163627 PMC8835888

[40] Tuntinarawat P Tangmanomana R Kittisiam T. Association between alteration of neutrophil to lymphocyte ratio, platelet to lymphocyte ratio, cancer antigen-125 and surgical outcomes in advanced stage ovarian cancer patient who received neoadjuvant chemotherapy. Gynecol Oncol Rep. (2024) 52:101347. 10.1016/j.gore.2024.101347 38419812 PMC10899061

